# Primary mitochondrial myopathy: Clinical features and outcome measures in 118 cases from Italy

**DOI:** 10.1212/NXG.0000000000000519

**Published:** 2020-10-20

**Authors:** Vincenzo Montano, Francesco Gruosso, Valerio Carelli, Giacomo Pietro Comi, Massimiliano Filosto, Costanza Lamperti, Tiziana Mongini, Olimpia Musumeci, Serenella Servidei, Paola Tonin, Antonio Toscano, Angela Modenese, Guido Primiano, Maria Lucia Valentino, Sara Bortolani, Silvia Marchet, Megi Meneri, Graziana Tavilla, Gabriele Siciliano, Michelangelo Mancuso

**Affiliations:** Department of Clinical and Experimental Medicine (V.M., F.G., G.S., M.M.), Neurological Clinic, University of Pisa, Italy; IRCCS Istituto delle Scienze Neurologiche di Bologna (V.C., M.L.V.), UOC Clinica Neurologica, Bologna, Italy; Department of Biomedical and Neuromotor Sciences (DIBINEM) (V.C., M.L.V.), University of Bologna, Italy; Dino Ferrari Centre (G.P.C.), Department of Pathophysiology and Transplantation (DEPT), University of Milan, Italy; Fondazione IRCCS Ca' Granda Ospedale Maggiore Policlinico (G.P.C., M.M.), Neuromuscular and Rare Disease Unit; Unit of Neurology (M.F.), ASST “Spedali Civili” and University of Brescia, Italy; UO Medical Genetics and Neurogenetics (C.L., S.M.), Fondazione IRCCS Istituto Neurologico C.Besta, Milan, Italy; Neuromuscular Unit (M.T., S.B.), Department of Neurosciences, University of Torino, Italy; Department of Clinical and Experimental Medicine (O.M., A.T., G.T.), UOC Neurologia e Malattie Neuromuscolari, University of Messina, Italy; UOC Neurofisiopatologia Fondazione Policlinico Universitario A. Gemelli IRCCS (S.S., G.P.), Roma, Italy; Dipartimento Universitario di Neuroscienze, Università Cattolica del Sacro Cuore (S.S., G.P.), Roma, Italy; Department of Neurosciences (P.T.), Biomedicine and Movement Sciences, Section of Clinical Neurology, University of Verona, Italy; Neurorehabilitation Unit (A.M.), Department of Neurosciences, University Hospital of Verona, Italy; Neuromuscular Unit (S.B.), Department of Neurosciences, University of Torino, Italy.

## Abstract

**Objective:**

To determine whether a set of functional tests, clinical scales, patient-reported questionnaires, and specific biomarkers can be considered reliable outcome measures in patients with primary mitochondrial myopathy (PMM), we analyzed a cohort of Italian patients.

**Methods:**

Baseline data were collected from 118 patients with PMM, followed by centers of the Italian network for mitochondrial diseases. We used the 6-Minute Walk Test (6MWT), Timed Up-and-Go Test (x3) (3TUG), Five-Times Sit-To-Stand Test (5XSST), Timed Water Swallow Test (TWST), and Test of Masticating and Swallowing Solids (TOMASS) as functional outcome measures; the Fatigue Severity Scale and West Haven-Yale Multidimensional Pain Inventory as patient-reported outcome measures; and FGF21, GDF15, lactate, and creatine kinase (CK) as biomarkers.

**Results:**

A total of 118 PMM cases were included. Functional outcome measures (6MWT, 3TUG, 5XSST, TWST, and TOMASS) and biomarkers significantly differed from healthy reference values and controls. Moreover, functional measures correlated with patients' perceived fatigue and pain severity. Patients with either mitochondrial or nuclear DNA point mutations performed worse in functional measures than patients harboring single deletion, even if the latter had an earlier age at onset but similar disease duration. Both the biomarkers FGF21 and GDF15 were significantly higher in the patients compared with a matched control population; however, there was no relation with severity of disease.

**Conclusions:**

We characterized a large cohort of PMM by evaluating baseline mitochondrial biomarkers and functional scales that represent potential outcome measures to monitor the efficacy of treatment in clinical trials; these outcome measures will be further reinvestigated longitudinally to define the natural history of PMM.

Mitochondrial disorders (MDs) are the most frequent metabolic deficits in human pathology and have a frequency of nuclear (nDNA) and mitochondrial DNA (mtDNA) pathogenic variants of 1:4,300.^1^ Myopathy may be the initial and isolated feature of MDs or may occur in association with other manifestations such as deafness, polyneuropathy, ataxia, stroke-like episodes, diabetes, and liver or kidney involvement.^2^ As recently proposed by an international consortium, “primary mitochondrial myopathies (PMM) are genetically defined disorders leading to defects of oxidative phosphorylation affecting predominantly, but not exclusively, skeletal muscle”.^3^

The age at onset, severity, and natural history of PMM are variable and range from serious early-onset forms with hypotonia, generalized weakness, and respiratory failure to late-onset milder forms with chronic progressive external ophthalmoplegia as the most common phenotype of PMM in adulthood.^3,4^

The same consortium has identified biomarkers and a series of clinical, functional, and quality of life scales to be applied to PMM, also in view of future pharmacologic studies.^3^

The primary objective of our study was the baseline clinical characterization of 118 patients with PMM using a set of measures and biomarkers, recruited from the Nation-wide Italian Collaborative Network of Mitochondrial Diseases.

## Methods

We prospectively collected clinical data, outcome measures, quality of life questionnaires, and biomarkers (ie, creatine kinase [CK], lactic acid, FGF21, and GDF15) in 118 adult Italian patients with a diagnosis of PMM due to either an mtDNA or nDNA mutation affecting oxidative phosphorylation in mitochondria, registered in the Nation-wide Italian Collaborative Network of Mitochondrial Diseases. Most of the enrolled patients were taking mitochondrial supplements (eg, coenzyme Q10, riboflavin, thiamine, and carnitine) at different dosages.

In a preliminary phase, a mailing list was created, and all PIs of the network analyzed the outcome measures proposed by the PMM experts' consortium^3^; after a deep e-discussion, several outcome measures and scales were identified:

### 

#### Clinician-reported outcome measures—clinical scales

The Newcastle Mitochondrial Disease Scale for Adults (NMDAS).

#### Functional tests

6-Minute Walk Test (6MWT), Timed Up-and-Go Test (x3) 3TUG, Five-Times Sit-To-Stand Test (5XSST), Timed Water Swallow Test (TWST), and Test of Masticating and Swallowing Solids (TOMASS).

#### Performance outcome measures

Spirometry.

#### Patient-reported outcome measures

Fatigue Severity Scale (FSS) and West Haven-Yale Multidimensional Pain Inventory (WHYMPI).

To reduce the intra- and intervariability among groups, all investigators met once for a face-to-face meeting, in which 3 facilitators (M.M., C.L., and O.M.) performed a detailed training on all adopted scales. Once the training had been completed, a specific CRF was developed, and 118 consecutive patients with PMM were enrolled and prospectively studied. Subjects were studied in the morning, and at the time of the visit, a fasting blood sample was taken for determination of CK, lactate, GDF15, and FGF21. In some patients, blood samples were either not collected or could not be completely processed for technical or methodological reasons.

FGF21 and GDF15 evaluation on patient serum was performed with Simple Plex cartridges using the Ella apparatus (ProteinSimple, San Jose) according to the manufacturer's instructions.

### Statistical analysis

Frequencies, average, median, SD, standard error, and percentiles were calculated for each feature. Values were reported as mean ± SD for variables with normal distribution, as median and interquartile range (IQR) for variables with skewed distribution, and as a percentage for categorical data. To verify the distribution of each parameter, the Kolmogorov-Smirnov test was performed. For continuous variables, the independent Student *t* test and Mann-Whitney *U* test were applied to find differences between 2 groups. Differences between results of functional tests, quality of life questionnaire scores, and laboratory values and their respective reference values in the healthy population were evaluated through the independent Student *t* test or Wilcoxon signed-rank test; reference values were derived from the literature.^5–12^ Continuous and categorical data were correlated through the Pearson correlation test or Spearman rank correlation test, according to the distribution of each parameter. To identify predictors of NMDAS results and quality of life questionnaire scores, univariate and multivariable logistic regression analysis was performed. Before performing partial correlations and regression analysis, variables with a skewed distribution were logarithmically corrected. Differences among patients harboring mtDNA mutation, mtDNA single deletion, and nDNA mutation were evaluated using analysis of variance one-way and Bonferroni post hoc tests for data with normal distribution and Kruskal-Wallis and Dunn-Bonferroni post hoc tests for data with skewed distribution, after assessing the equality of variances for each variable using parametric and nonparametric Levene tests. A similar analysis was conducted by dividing the patients into those with defective mitochondrial translation machinery, with mtDNA deletions, and with defects in respiratory chain (RC) subunits/assembly factors. For analysis on TOMASS scores among patients harboring different mutation types, the Mann-Whitney *U* test was performed between all pairs of groups because variances resulted heterogeneous, and values were skewed distributed. In all cases, a *p* value of less than 0.05 was regarded as significant; a lower value was indicated if it was found. Biostatistical analysis was performed with IBM SPSS 20.0.0 program.

### Data availability

Current article data are accessible from Michelangelo Mancuso, University of Pisa. In accordance with the data protection legislation in Europe (General Data Protection Regulation), to share the data of the Italian Network, it is necessary to stipulate an agreement between the University of Pisa and the applicant institution. Study data can be requested by contacting Michelangelo Mancuso (michelangelo.mancuso@unipi.it).

### Standard protocol approvals, registrations, and patient consents

Written informed consent was obtained from all participants, and the ethics committees of each center approved the study.

## Results

A total of 118 PMM cases were enrolled (50 men), with mean age 50.62 ± 12.6 years, disease onset 31.40 ± 14.7 years, and disease duration 21.5 ± 12.3 years.

Table 1 shows the whole data set as mean ± SD or median and IQR, according to the distribution of each value (normal or skewed distribution). Functional test results (6MWT, 3TUG, 5XSST, TWST, and TOMASS) and questionnaire results (FSS and WHYMPI) were compared with the respective normal values, differentiated by sex for the TOMASS and considering the lower limit of normality for FEV1.

**Table 1:**
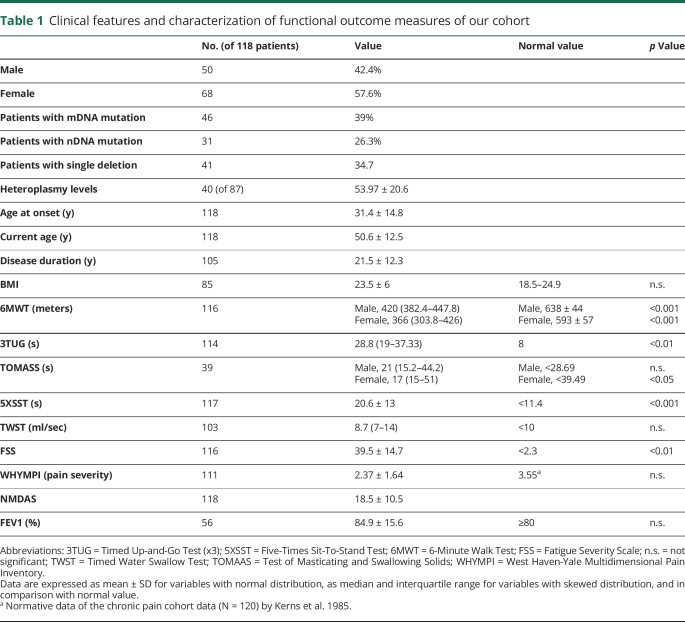
Clinical features and characterization of functional outcome measures of our cohort

Forty-six patients (39%) harbored an mtDNA point mutation, 31 (26.3%) a nuclear gene mutation, and 41 (34.7%) an mtDNA single deletion. Genotypes are shown in supplementary table 1, links.lww.com/NXG/A325, categorized as done by Lethonen^13^ into defective mitochondrial translation machinery, mtDNA deletions, and defects of RC subunits/assembly factors. In 12 patients with mtDNA multiple deletions, at the time of the submission, WES is still ongoing (already tested for mtDNA point mutations, *POLG*, *ANT1, Twinkle*, and *OPA1*). Heteroplasmy levels, available from muscle specimens in 40 patients (46%), were not significantly different between mtDNA point mutation and single deletion groups. Heteroplasmy levels obtained in other tissues (blood or urine sediment) were not considered to reduce bias in statistic comparisons.

**Figure 1:**
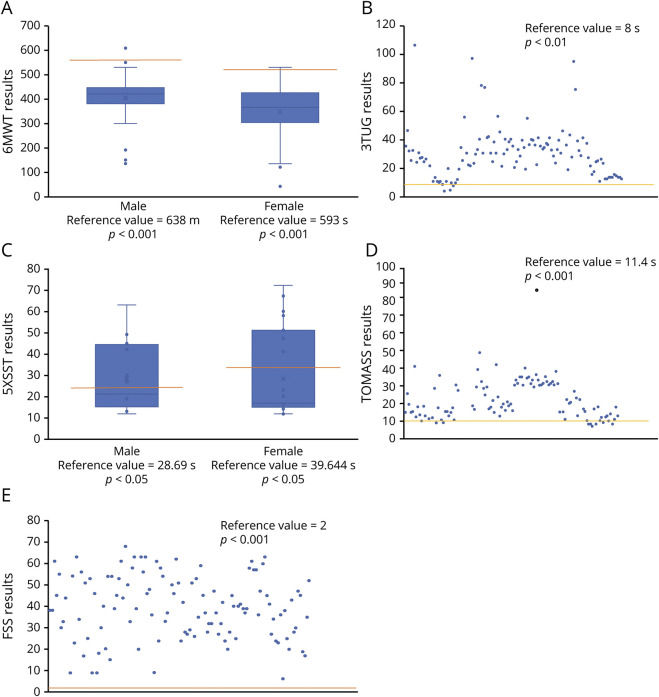
Results (A–D) Composite figure showing functional test results. (A): 6MWT values in males and females; (B): 3TUG result distribution; (C): 5XSST result distribution; (D): TOMASS result distribution; (E): FSS result distribution. 3TUG = Timed Up-and-Go Test (x3); 5XSST = Five-Times Sit-To-Stand Test; 6MWT = 6-Minute Walk Test; FSS = Fatigue Severity Scale; TOMASS = Test of Masticating and Swallowing Solids.

We observed a significant difference for the 6MWT (*p* < 0.001, figure 1A), 3TUG (*p* < 0.01, figure 1B), 5XSST (*p* < 0.001, figure 1C), TOMASS (only in female *p* < 0.05, figure 1D), and FSS (*p* < 0.01, figure 1E) compared with normative values. FEV1 resulted in the range of normality (table 1).

The WHYMPI gives an overall estimation of the pain experience, taking into account cognitive and behavioral factors, with a direct correlation between the WHYMPI score and the severity of pain. The 52 WHYMPI items are divided into 3 sections, with part I subitems evaluating pain severity. PMM cases showed high scores for pain severity even when compared with the 120 chronic pain cohort data by Kerns et al.^11^ (table 1), in which most patients (81.5%) were male (vs 42% of our cohort) but with the same mean age at the evaluation (50.8 vs 50.6 years of our study).

**Figure 2:**
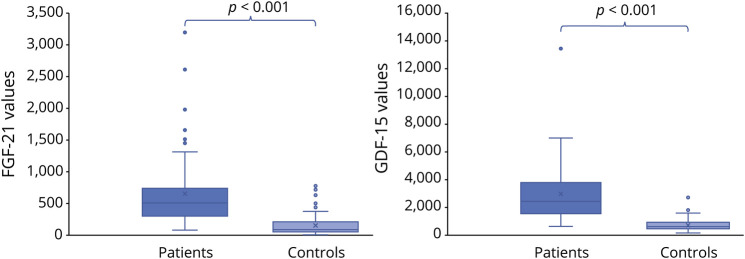
FGF21 and GDF15 values in patients and controls

Both exploratory mitochondrial biomarkers FGF21 and GDF15, available in 80 patients, were significantly higher (*p* < 0.001) when compared with a matched control population (table 2 and figure 2). On the contrary, blood lactate at rest and CK did not significantly differ between patients with PMM and healthy controls; however, lactate was higher than 1.8 mMol/L in 54.3% of patients (and above 2 mMol/L in 30.5%), and CK values were slightly altered in 34.4% of men and 33.3% of women. Blood biomarkers were not correlated with genotypes, disease duration, FSS, NMDAS, and TWST. Exploratory biomarkers were not significantly different between male and female patients. CK showed a trend toward statistical significance (*p* = 0.059), being higher in men.

**Table 2:**
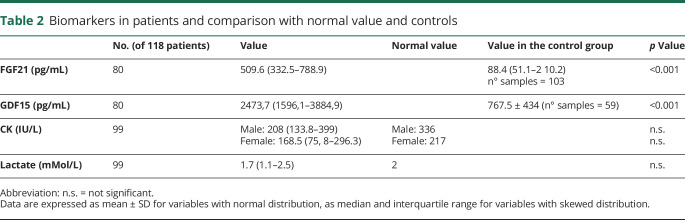
Biomarkers in patients and comparison with normal value and controls

**Figure 3:**
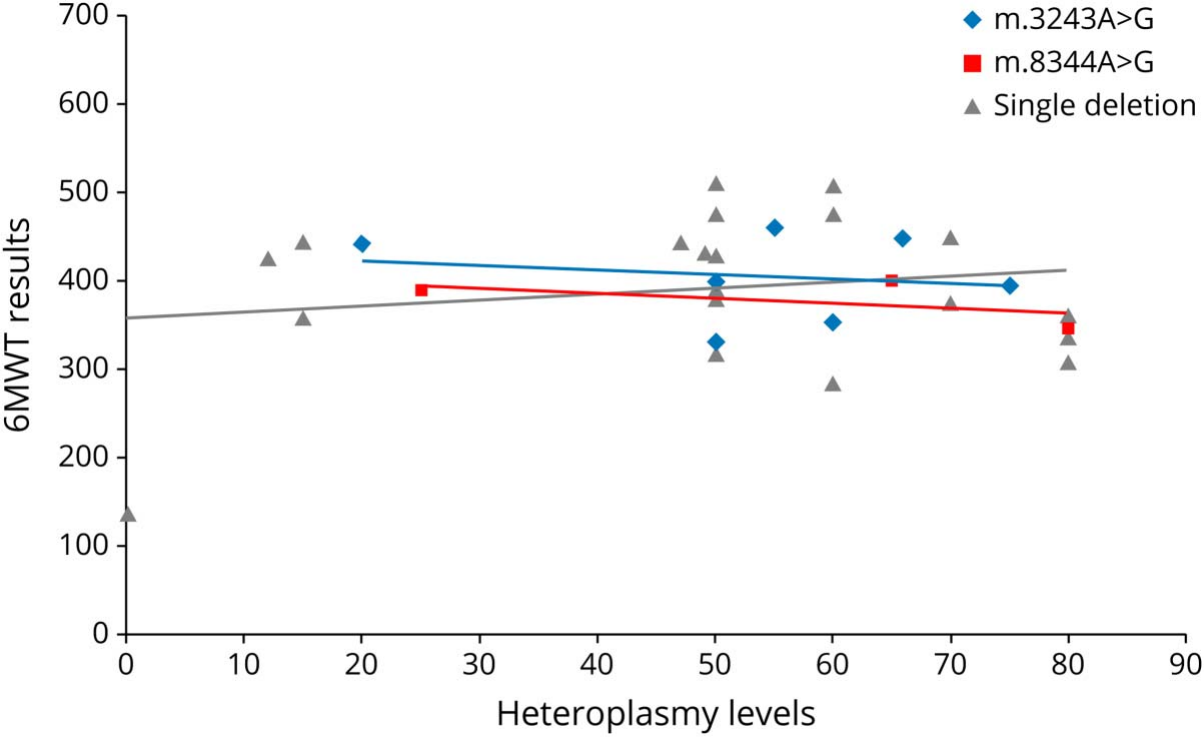
Correlation between heteroplasmy levels and 6MWT results 6MWT = 6-Minute Walk Test.

Heteroplasmy levels in muscle did not correlate with clinical features or functional tests or scale severity. Figure 3 shows, as an example, the relationship between the 6MWT and the most common mtDNA genotypes in our cohort; the same is observed with all other parameters (data not shown). Similarly, no differences were observed comparing heteroplasmy levels between the mtDNA mutation and single deletion groups and functional tests or scales. A subanalysis was conducted on the 20 m.3243A>G patients; lactate was elevated in 40% of these patients. Mitochondrial biomarkers (FGF21 and GDF15) were available in 15/20; in this subset of patients, lactate levels (but not CK) correlated with FGF21, GDF15, FEV1, and heteroplasmy levels, but not with functional test scores.

We also evaluated the differences among the PMM genotypes (mtDNA mutation, mtDNA single deletion, and nDNA mutation). Patients harboring single deletions performed significantly better than others in the 6MWT, 3TUG, and 5XSST and had an earlier age at onset. However, they showed the worst results in the TOMASS and FEV1. The mtDNA and nDNA mutant patients did not differ significantly on functional scales and showed worst performances than single deletion (supplementary table 2, links.lww.com/NXG/A325).

Moreover, a bivariate analysis showed a direct correlation between the 6MWT and males, between FEV1 and females, NMDAS and disease duration (table 3). The observed differences in pain severity between males and females could be an expression of hormonal factors^6^; no difference was observed in biomarker levels.

**Table 3:**
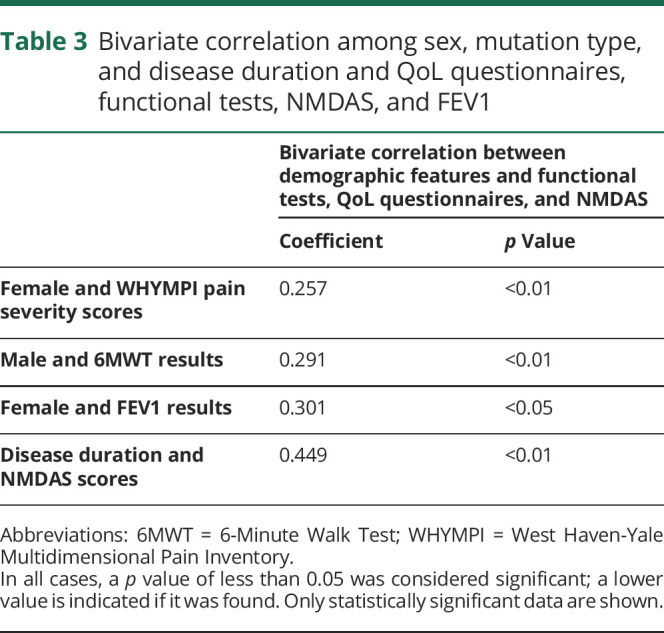
Bivariate correlation among sex, mutation type, and disease duration and QoL questionnaires, functional tests, NMDAS, and FEV1

A correlation analysis among the functional scales was also conducted. It revealed an indirect correlation between the 6MWT and FSS, between the 6MWT and NMDAS, and a direct correlation between 3TUG and TWST and FSS and NMDAS scores (Supplementary table 3, links.lww.com/NXG/A325). According to the relations observed among gender, mutation type, disease duration and clinical, functional tests, and QoL questionnaires scores, we performed a correlation and multivariate regression analysis, including gender, mutation type, and duration of disease as independent variables. This multivariate analysis model confirmed the relation between functional scales and NMDAS and FSS results, regardless of the other features of patients (Supplementary table 4, links.lww.com/NXG/A325). Therefore, FSS indirectly correlates with the 6MWT and directly with the other scales, thus revealing an agreement between the fatigue perceived by the patient and the objective motor functional scales. Finally, a correlation between the NMDAS and the motor functional scales was observed.

Patients were also analyzed by considering the subgroups identified by Lethonen (supplementary table 4, links.lww.com/NXG/A325).^13^ Among these subgroups, there were no statistically significant differences in clinical features, functional tests, or biomarkers, except for RC subunit–mutated patients (only 3 patients, no statistical power) who showed an earlier age at onset and higher pain severity. The only RC-mutated patient with exploratory biomarker available showed low FGF21 and GDF15 values.

Bivariate correlation among mitochondrial biomarkers, QoL questionnaires, functional tests, NMDAS, and FEV1 were conducted separately in the subgroups according to Lethonen (supplementary table 5, links.lww.com/NXG/A325), excluding the RC-mutated patients because poorly represented. In mitochondrial translation machinery–mutated patients, FGF21 and GDF15 values directly correlated with heteroplasmy levels, and GDF15 and lactate directly correlated with NMDAS. In mtDNA deletions, GDF15 directly correlated with lactate.

## Discussion

We report a cohort study describing the baseline features of 118 adult patients with PMM, in which several outcome measures suggested by the International Consortium^3^ were assessed.

Data about 6MWT results in our cohort were compared with the ones presented in the studies of Karaa et al.^14^ and Madsen et al.^15^ Although we observed similar results in the 6MWT between our cohort and the MOTOR study,^15^ a significant difference (*p* < 0.01) between our 6MWT values and the ones in MMPOWER^14^ was observed. A possible explanation could be that MMPOWER enrolled more females (83%) compared with MOTOR (58%) and our study (57.6%).

FGF21 and GDF15 levels were significantly altered compared with the healthy control group. Although we did not find any difference in FGF21 levels between PMM genotypes, we confirm the role of FGF21 as a reliable serum marker in mitochondrial disease diagnostics, whereas lactate and CK levels are not. In the subgroup with defective mitochondrial translation machinery, FGF21 and GDF15 directly correlated with heteroplasmy levels and disease severity as valued by NMDAS.

It is notable that there were no significant differences of CK and lactate levels between patients with PMM and normal values, whereas there were for FGF21 and GDF15. We confirm that normal CK and lactate values, being altered only in a small portion of patients with specific genotypes, do not exclude the diagnosis of PMM as well as other metabolic myopathies.^1,16^

Clinically, this could be very helpful in general neurologic settings—where these patients are likely to complain of pain, myalgia, cramps, reduced exercise tolerance, common and nonspecific complaints in PMM. In this context, CK and lactate screening could lead to a false-negative diagnosis of PMM, whereas FGF21 and GDF15 do not. However, CK and lactate still play an important role in the patient's follow-up, especially in those cases associated with lactic acidosis or rhabdomyolysis.

GDF15 was also elevated in our patients, but a wide variety of other neuromuscular diseases of nonmitochondrial origin (such as muscular dystrophy or spinal muscular atrophy) can induce high GDF15 levels; indeed, GDF15 has higher sensitivity but lower specificity.^17–19^ Measurement of FGF21 and GDF15 concentrations in serum might be useful as a first-line diagnostic tool for PMM, whereas their role as a potential biomarker for therapeutic efficacy in future trials is still unclear. In a recent trial involving deoxynucleoside therapy in thymidine kinase 2–mutated PMM, Domínguez-González et al.^20^ promisingly showed a significant decrease in GDF15 levels after treatment. Additional data and longitudinal studies are required to validate their utility in clinical trials and to determine whether they can be used as proof of clinical efficacy of a treatment.

The functional tests and scales adopted here were impaired in PMM. This confirms that they could represent useful tools to monitor disease status and also play a potential role as outcome measures to evaluate the effectiveness of drugs in upcoming clinical trials. Moreover, the functional scales showed a strong agreement with the perceived fatigue evaluated with the FSS and pain severity assessed by the WHYMPI. Therefore, they represent good, reliable, and easily assessable measures of fatigue, pain, and exercise intolerance, common symptoms and complaints of PMM that are often difficult to measure in clinical practice.^4^

The 6MWT is a well-validated outcome measure for a large group of neuromuscular diseases and is also used to monitor the natural history and the efficacy of treatments.^21–23^ Consistently, the 6MWT is a good measure of motor impairment in PMM, which correlates with the perceived exercise intolerance, pain severity, and fatigue. However, in view of future therapeutic trials, there are no data on the 6MWT decline over time in PMM. Thus, longitudinal observational studies are strongly needed in PMM.

The 3TUG is noninvasive and easy to perform in daily practice and provides a quick score of pelvic girdle weakness and fatigue; it has recently been tested in Lambert-Eaton myasthenia and is sensitive to 3,4-diaminopyridine therapy.^24^ The 3TUG and 5XSST in our cohort were significantly elevated, and the 3TUG correlated well with fatigue and pain severity.

Furthermore, an indirect correlation was found between the NMDAS score and functional performance (6MWT, 3TUG, and TWST), thus confirming the role of NMDAS as the referred scale for clinic evaluation also in PMM. The correlation between the NMDAS score and disease duration seems to suggest a worse clinical phenotype in patients with earlier diagnosis.

Patients with different mutations also performed differently in functional scales, whereas genotype did not influence disease duration and NMDAS scores. Single deletion, with an earlier age at onset, seemed to perform better in the 6MWT, 3TUG, and 5XSST, but had a worse score result in the TOMASS and FEV1, whereas mtDNA point mutations and nDNA-mutated patients showed worse results in the 6MWT, 3TUG, and 5XSST. Of interest, heteroplasmy did not correlate with functional tests or scales. However, heteroplasmy levels were available only in half of our mtDNA-related population, and this lack of correlation should be further explored in additional and even larger cohorts. In m.3243A>G patients, although we have found a significant correlation between lactate and FGF21, GDF15, FEV1, and heteroplasmy levels, we failed to confirm the correlation between heteroplasmy levels and disease severity, as found in the UK population.^25^

FSS and WHIMPI appeared skewed toward the female gender, without any significant difference in FGF21 or GDF15. This could be due to hormonal factors or difference in muscle mass because CK and BMI showed higher values in males.^16,26^

Swallowing impairment is frequently observed in PMM,^27,28^ in about 15% of Italian patients with PEO,^27^ with females being affected more, probably because of more severe weakness, as already reported.^29^ Dysphagia also seems to be a prominent feature in single deletion patients; compared with other genotypes, we found significant worst scores in the swallowing scale in single deletion patients, thus confirming that dysphagia is a canonical feature of this disorder.^30,31^

Therefore, we confirm the need for a periodic phoniatric assessment of swallowing, considering the disease burden, morbidity, and mortality associated with ab ingestis pneumonia. FEV1 in our cohort tended to the upper than the lower level of normality. However, it would be advisable to reevaluate the spirometry of these patients over time because patients with PMM may develop respiratory failure, and it is not known how and with what severity it manifests over time. Although we did not find any significant alteration in the respiratory measure, we stress the necessity of periodic evaluation of pulmonary function tests and spirometry.

Our study has some limitations. We have enrolled patients at different stages of disease, and the timing of the biomarkers analysis and of the clinical analysis was variable from patient to patient, and some subset of outcome measures selected are not available for all 118 participants. In 12 patients with mtDNA multiple deletions, the nuclear defect is still unknown; however, we believe that this does not affect the main conclusions of our study. Moreover, functional outcomes are highly dependent on the skill of the investigator and very sensitive to interindividual variance. To reduce the inter-intra variability, training sessions were organized before the study, and the same investigators were involved in all measurements. In addition, we did not collect control values—beside FGF21 and GDF15—but we have referred to normative values where N size, sex, and age distribution of the control data may be not clearly stated. However, our final goal was to obtain a clear picture of basal values in PMM as a starting baseline in view of natural history studies and clinical trials. Finally, we have collected these cross-sectional data at time 0, but it is crucial to also monitor them during the natural history of the PMM (follow-up is ongoing at our centers).

We characterized a large cohort of patients with PMM, providing baseline data on a functional scale that could be reliable functional outcome measures in future PMM clinical trials (6MWT, 3TUG, 5XSST, TWST, and TOMASS). Functional test results fit well with perceived fatigue and pain, and elevated FGF21 and GDF15 distinguished patients with PMM from healthy controls. Further investigations, evaluating the longitudinal evolution of this outcome measures over time, are strongly needed and planned to understand PMM natural history to quantify the impact of disease-modifying therapies.

## Glossary

3TUG: Timed Up-and-Go Test (x3)
5XSST: Five-Times Sit-To-Stand Test
6MWT: 6-Minute Walk Test
CK: creatine kinase
FSS: Fatigue Severity Scale
IQR: interquartile range
MD: mitochondrial disorder
mtDNA: mitochondrial DNA
NMDAS: The Newcastle Mitochondrial Disease Scale for Adults
PMM: primary mitochondrial myopathy
RC: respiratory chain
TOMAAS: Test of Masticating and Swallowing Solids
TWST: Timed Water Swallow Test
WHYMPI: West Haven-Yale Multidimensional Pain Inventory
